# Supplementary material for: PMI estimation through metabolomics and potassium analysis on animal vitreous humour
Source: Int J Legal Med. 2023 Feb 17;137(3):887–95. doi: 10.1007/s00414-023-02975-6 (PMC10085955; doi:10.1007/s00414-023-02975-6)
Supplement: Supplementary file 1 — Supplementary Table S1. List of ovine VH metabolites quantified using the Chenomx Profiler tool. Fig. S1 Values of PMI predicted for the test set (PMI predicted) vs measured values (PMI): predictions from the model obtained considering the quantified metabolites (panel A), the vitreous potassium concentration (panel B) and the combination of quantified metabolites and potassium concentration (panel C); the diagonal is reported in the plots. Fig. S2 Profiles of the significantly relevant metabolites discovered by both ordinal and regression models: 3-hydroxybutyrate (panel A), alanine (panel B), glutamate (panel C) and glycine (panel D); orthogonal scaling has been applied to concentration to have the same scale [-1,1] for all the metabolites; dashed lines indicate the linear regression lines [file 414_2023_2975_MOESM1_ESM.docx]

**PMI estimation through metabolomics and potassium analysis on animal vitreous humour**

Emanuela Locci^1^, Matteo Stocchero^2^, Rossella Gottardo^3^, Alberto Chighine^1,^ *, Fabio De-Giorgio^4, 5^, Giulio Ferino^1^, Matteo Nioi^1^, Roberto Demontis^1^, Franco Tagliaro^3^, Ernesto d’Aloja^1^

^1^Department of Medical Sciences and Public Health, Section of Legal Medicine, University of Cagliari, Cagliari, Italy.

^2^Department of Women’s and Children’s Health, University of Padova, Padova, Italy.

^3^Department of Diagnostics and Public Health, Unit of Forensic Medicine, University of Verona, Verona, Italy.

^4^Department of Health Surveillance and Bioethics, Section of Legal Medicine, Catholic University of Rome, Rome, Italy.

^5^Fondazione Policlinico Universitario A. Gemelli IRCCS, Rome, Italy.

*Corresponding author: Alberto Chighine [alberto.chighine@unica.it](mailto:alberto.chighine@unica.it)

**Supplementary Table S1.** List of ovine VH metabolites quantified using the Chenomx Profiler tool.

| **Compound** | **PubChem (CID)** |
| --- | --- |
| 2-Hydroxybutyrate | 440864 |
| 3-Hydroxybutyrate | 354112 |
| 3-Hydroxyisobutyrate | 11966314 |
| Acetate | 175 |
| Acetone | 180 |
| Alanine | 5950 |
| Allantoin | 204 |
| Ascorbate | 54670067 |
| Aspartate | 5960 |
| Betaine | 247 |
| Carnitine | 288 |
| Choline | 305 |
| Creatine | 586 |
| Creatinine | 588 |
| Dimethylsulfone | 6213 |
| Dimethylamine | 674 |
| Ethanol | 702 |
| Ethanolamine | 700 |
| Formate | 283 |
| Fumarate | 5460307 |
| Glucose | 5793 |
| Glutamate | 33032 |
| Glutamine | 5961 |
| Glutathione | 124886 |
| Glycerol | 753 |
| Glycine | 750 |
| Hypoxanthine | 135398638 |
| Inosine | 135398641 |
| Isoleucine | 6306 |
| Lactate | 91435 |
| Leucine | 6106 |
| Lysine | 5962 |
| Methanol | 887 |
| N-Acetylaspartate | 65065 |
| N-Acetylglucosamine | 24139 |
| Nicotinurate | 68499 |
| O-Acetylcarnitine | 7045767 |
| O-Phosphocholine | 1014 |
| Ornithine | 6262 |
| Phenylalanine | 6140 |
| Pyruvate | 107735 |
| Serine | 5951 |
| Succinate | 160419 |
| Taurine | 1123 |
| Threonine | 6288 |
| Trimethylamine | 1146 |
| Tryptophan | 6305 |
| Tyrosine | 6057 |
| Uracil | 1174 |
| Valine | 6287 |
| myo-Inositol | 892 |
| π-Methylhistidine | 92105 |

**Fig. S1** Values of PMI predicted for the test set (PMI predicted) vs measured values (PMI): predictions from the model obtained considering the quantified metabolites (panel A), the vitreous potassium concentration (panel B) and the combination of quantified metabolites and potassium concentration (panel C); the diagonal is reported in the plots.


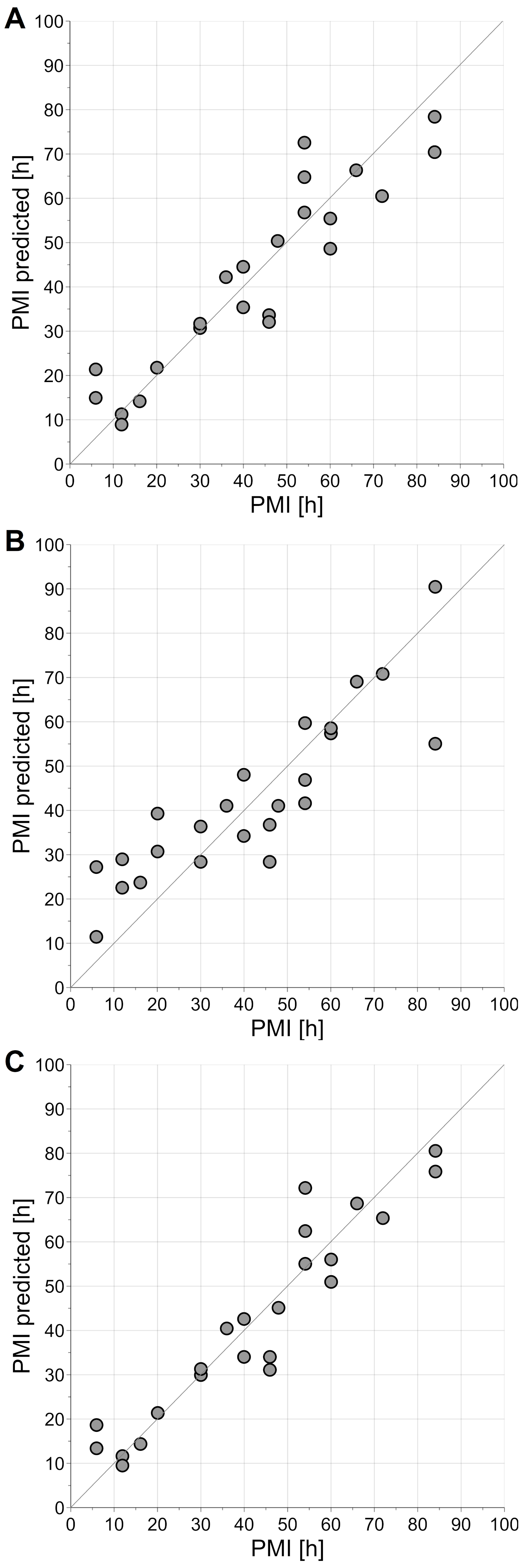


**Fig. S2** Profiles of the significantly relevant metabolites discovered by both ordinal and regression models: 3-hydroxybutyrate (panel A), alanine (panel B), glutamate (panel C) and glycine (panel D); orthogonal scaling has been applied to concentration to have the same scale [-1,1] for all the metabolites; dashed lines indicate the linear regression lines

**
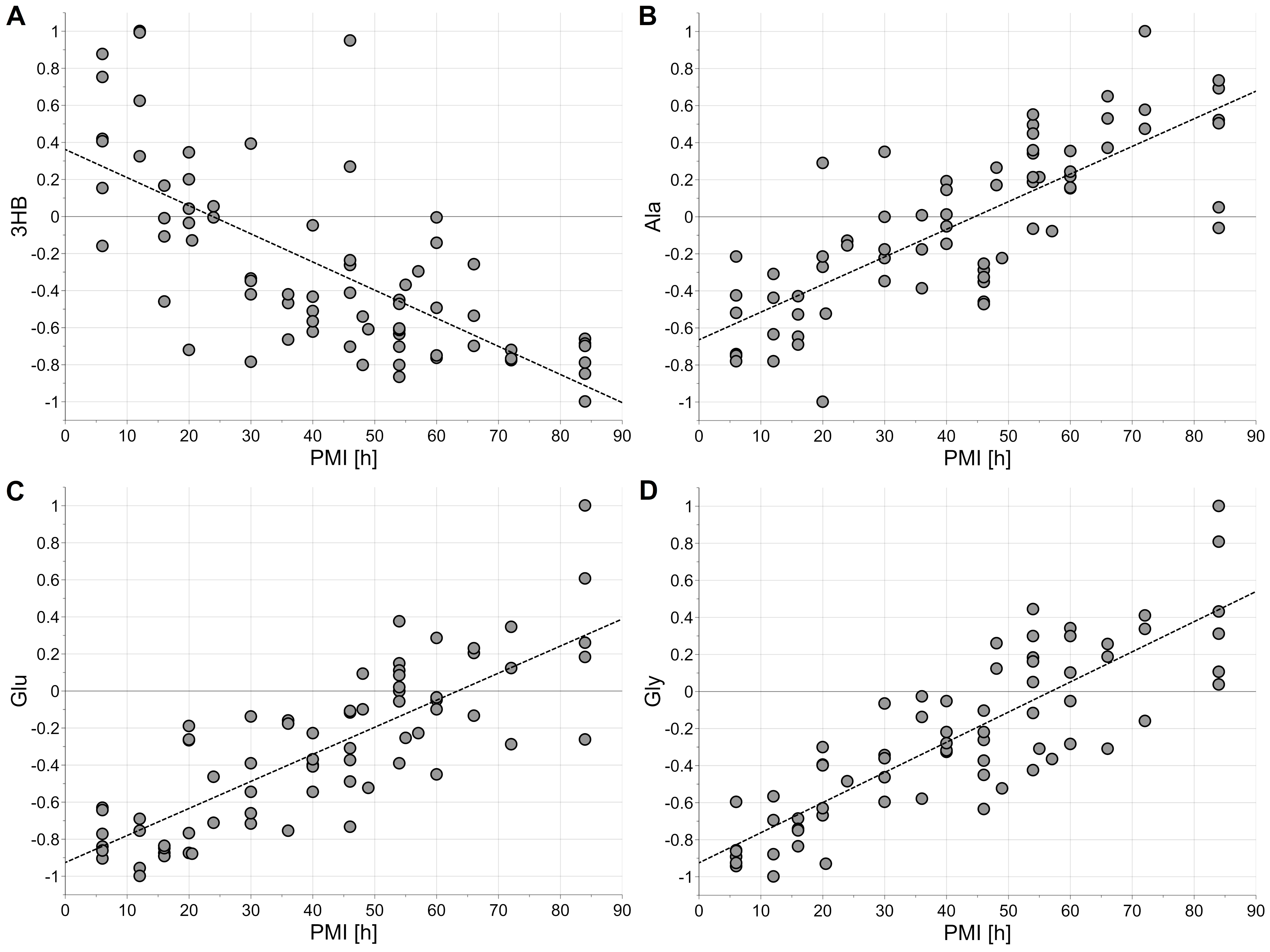
**
